# Deterioration of the fixation segment’s stress distribution and the strength reduction of screw holding position together cause screw loosening in ALSR fixed OLIF patients with poor BMD

**DOI:** 10.3389/fbioe.2022.922848

**Published:** 2022-08-30

**Authors:** Jing-Chi Li, Zhi-Qiang Yang, Tian-Hang Xie, Zhe-Tao Song, Yue-Ming Song, Jian-Cheng Zeng

**Affiliations:** ^1^ Department of Orthopedic Surgery and Orthopedic Research Institute, West China Hospital/West China School of Medicine for Sichuan University, Chengdu, China; ^2^ Department of Imaging, West China Hospital, Chengdu, China

**Keywords:** oblique lumbar interbody fusion, screw loosening, biomechanical deterioration, anterior lateral single rod fixation, screw holding plane, stress distribution

## Abstract

The vertebral body’s Hounsfield unit (HU) value can credibly reflect patients’ bone mineral density (BMD). Given that poor bone-screw integration initially triggers screw loosening and regional differences in BMD and strength in the vertebral body exist, HU in screw holding planes should better predict screw loosening. According to the stress shielding effect, the stress distribution changes in the fixation segment with BMD reduction should be related to screw loosening, but this has not been identified. We retrospectively collected the radiographic and demographic data of 56 patients treated by single-level oblique lumbar interbody fusion (OLIF) with anterior lateral single rod (ALSR) screw fixation. BMD was identified by measuring HU values in vertebral bodies and screw holding planes. Regression analyses identified independent risk factors for cranial and caudal screw loosening separately. Meanwhile, OLIF with ALSR fixation was numerically simulated; the elastic modulus of bony structures was adjusted to simulate different grades of BMD reduction. Stress distribution changes were judged by computing stress distribution in screws, bone-screw interfaces, and cancellous bones in the fixation segment. The results showed that HU reduction in vertebral bodies and screw holding planes were independent risk factors for screw loosening. The predictive performance of screw holding plane HU is better than the mean HU of vertebral bodies. Cranial screws suffer a higher risk of screw loosening, but HU was not significantly different between cranial and caudal sides. The poor BMD led to stress concentrations on both the screw and bone-screw interfaces. Biomechanical deterioration was more severe in the cranial screws than in the caudal screws. Additionally, lower stress can also be observed in fixation segments’ cancellous bone. Therefore, a higher proportion of ALSR load transmission triggers stress concentration on the screw and bone-screw interfaces in patients with poor BMD. This, together with decreased bony strength in the screw holding position, contributes to screw loosening in osteoporotic patients biomechanically. The trajectory optimization of ALSR screws based on preoperative HU measurement and regular anti-osteoporosis therapy may effectively reduce the risk of screw loosening.

## 1 Introduction

Anterior lateral single rod (ALSR) fixation can provide sufficient instant postoperative stability for oblique lumbar interbody fusion (OLIF) patients without the need for other surgical incisions (Zhao et al., 2022a; Zhao et al., 2022b). As a hardware-related complication, screw loosening has been widely reported, negatively affecting patients’ rehabilitation and deteriorating long-term prognosis (Bokov et al., 2019; Zou et al., 2020). Osteoporosis is an essential risk factor for this complication. Bone-screw integration was aggravated with the reduction in bone mineral density (BMD); this was proven to be the primary mechanism for the higher risk of screw loosening in osteoporotic patients (Bokov et al., 2019; Zou et al., 2020).

Traditionally, the dual-energy X-ray absorptiometry is the gold standard for diagnosing osteoporosis. However, this imaging examination cannot eliminate pathological bone formation during lumbar degenerative diseases (e.g., osteophytes, endplate sclerosis, and zygapophyseal joint osteoarthritis). This leads to an underestimation of the severity of osteoporosis in patients with lumbar degenerative diseases (Mikula et al., 2019; Zou et al., 2020). The vertebral Hounsfield unit (HU) value measured by computed tomography (CT) has been widely used to diagnose osteoporosis (Bredow et al., 2016; Gausden et al., 2017). The confounding effect of pathological bone formation can be eliminated during the measurement of HU in the vertebral body by adjusting the region of interest (Mi et al., 2017; Zou et al., 2019). Thus, HU has become a credible indicator in BMD judgment.

**FIGURE 1:**
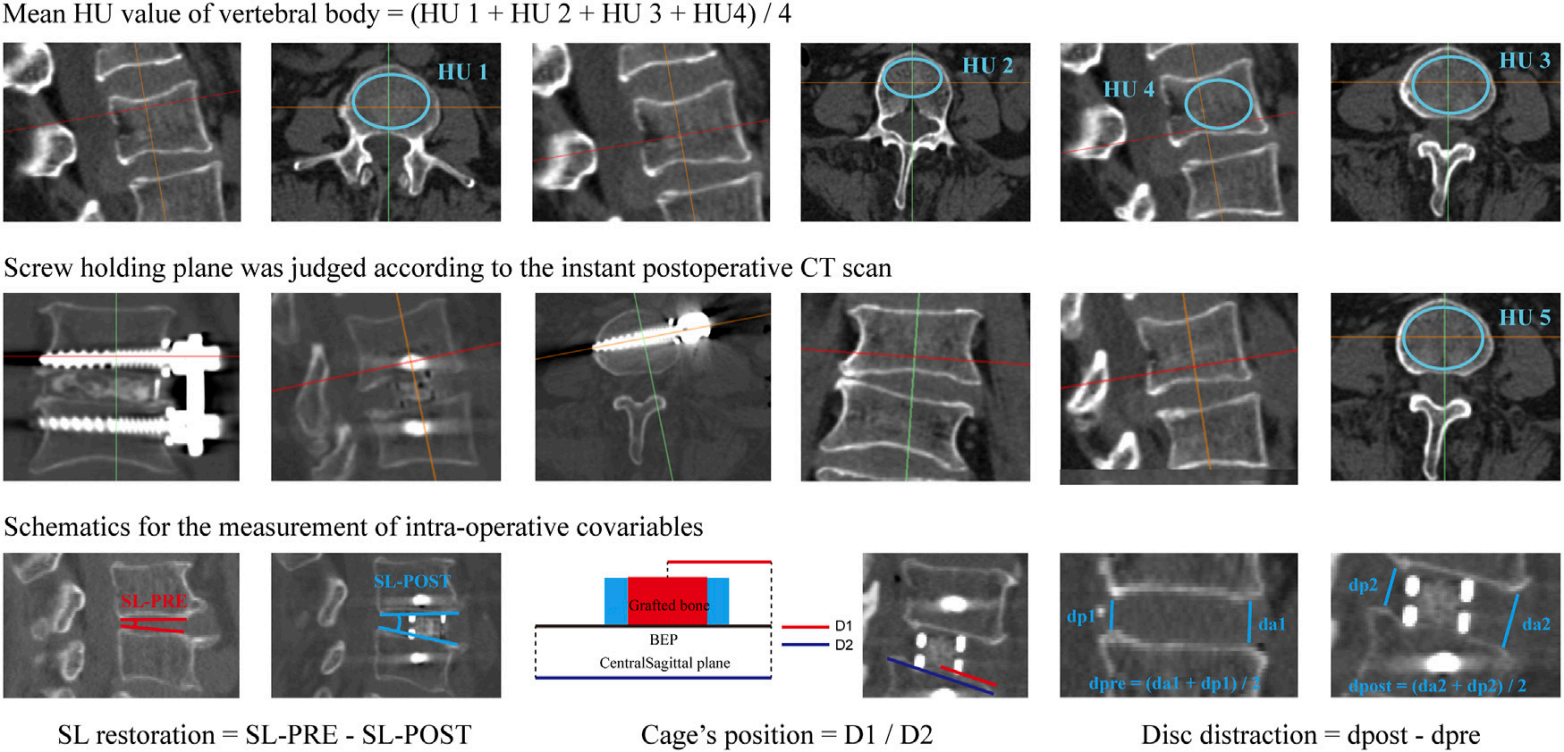
Schematic of different HU measurement methods and the measurement of intraoperative covariables. HU1, HU in the transverse plane close to the superior BEP; HU2, HU in the central transverse plane; HU3, HU in the transverse plane close to the inferior BEP; HU4, HU in the midsagittal plane; The mean value of HU1 to HU4 was defined as the mean HU of the vertebral body; HU5, HU value of the screw holding plane.

Presently, the HU value of the vertebral body is defined by the average value of four planes, including the midsagittal plane, central transverse plane, and transverse planes close to the superior and inferior bony endplates (Figure 1) (Bredow et al., 2016; Zou et al., 2019). Although this HU definition method is commonly used in BMD judgment and screw loosening risk prediction for patients with lumbar screw fixation, it still has inherent defects: it cannot directly reflect the BMD in the screw holding plane. As mentioned above, the yield strength reduction of cancellous bone is the main biomechanical mechanism for poor bone-screw integration and resulting screw loosening in osteoporotic patients, and regional differences in BMD and strength in cancellous bone exist (Smit et al., 1997; Wegrzyn et al., 2010). We hypothesize that the HU measurement of the screw holding plane can better reflect changes in these local effects.

As above mentioned, surgeons believe that the decreased bony strength is the main reason for the increased risk of screw loosening in osteoporotic patients. Meanwhile, studies illustrated that stress concentration on the bone-screw interfaces and fixation screws would aggravate poor bone-screw integration and result in screw loosening (Tsuang et al., 2016; Pearson et al., 2017; Nowak, 2019; Kanno et al., 2021). Specifically, according to the stress shielding effect, the reduction of BMD will aggravate the stiffness differences between bony structures and titanium screws (Agarwal et al., 2016; Hsieh et al., 2020). As a result, a higher proportion of stress should be transported by the screw fixation system. Therefore, we hypothesize that this may be the potential mechanism for the stress concentration of screw and bone screw interfaces. In other words, a higher risk of screw loosening in osteoporotic patients may not be limited to poor bone quality but also biomechanical deterioration in bone-screw interfaces, but this has still not been verified.

In this study, to verify these hypotheses, we investigated whether the HU in the screw holding plane is a better predictor during the judgment of screw loosening and investigated changes in the load transmission proportion between the vertebral body and ALSR screw system with BMD stepwise reduction. The prospectively collected radiographic and demographic data of OLIF patients fixed by ALSR were retrospectively reviewed. Changes in the stress distribution of the ALSR fixation segment were investigated by computing biomechanical changes in fixation screws, bone-screw interfaces, and cancellous bones of vertebral bodies in an anteriorly constructed and validated lumbosacral model. This study could provide theoretical guidance for understanding the screw loosening mechanism and feasible methods to reduce the risk of screw loosening.

## 2 Materials and methods

### 2.1 Review of prospectively collected radiographic and demographic data

#### 2.1.1 Patient collection

The ethics committees of West China Hospital approved the protocol of this study (2020-554). Informed consent was waived for this retrospective study. We retrospectively reviewed the radiographic and demographic data of OLIF patients with ALSR screw fixation from May 2017 to August 2019. Their age, sex, and body mass index (BMI) were recorded. A senior spine surgeon performed all operations. Screw types and sizes were identical in these patients. All screws were placed in a single attempt and penetrated the contralateral cortex.

Patients who underwent single segment OLIF with ALSR screw fixation for patients with lumbar degenerative diseases, including spinal stenosis, grade 1 and grade 2 degenerative spondylolisthesis, and lumbar disc herniation, were included in this study. The exclusion criteria were as follows: 1) Patients with a history of lumbar surgery; 2) Patients with primary or metastatic spinal tumors, lumbar tuberculosis, rheumatic immune diseases, and secondary osteoporosis caused by medication or other metabolic diseases; 3) Patients with grade 3 and grade 4 degenerative spondylolisthesis or spondylolysis; 4) Patients who underwent lumbar revision surgery within the clinical follow-up period of 12 months for complications other than screw loosening; 5) Patients who underwent intraoperative screw replacement.

#### 2.1.2 Radiographic data collections

Patients underwent lumbar computational tomography (CT) three times in the imaging center of West China Hospital, including 1 week before, 1 week after, and 1 year after OLIF surgery. The tube voltage was set to 120 kV, and all CT scan setting parameters were uniform in all enrolled patients (Mikula et al., 2019; Xi et al., 2020; Zou et al., 2020). An experienced spine surgeon independently measured the screw loosening status and radiographic parameters mentioned in the Figure 1. The interobserver and intraobserver reliability of these measured parameters was verified in 10 randomly selected patients. One week after the imaging measurement, the spine surgeon and a senior radiologist independently remeasured the imaging parameters of these selected patients. These measurement results were recorded separately to verify intraobserver and interobserver consistency.

**FIGURE 2:**
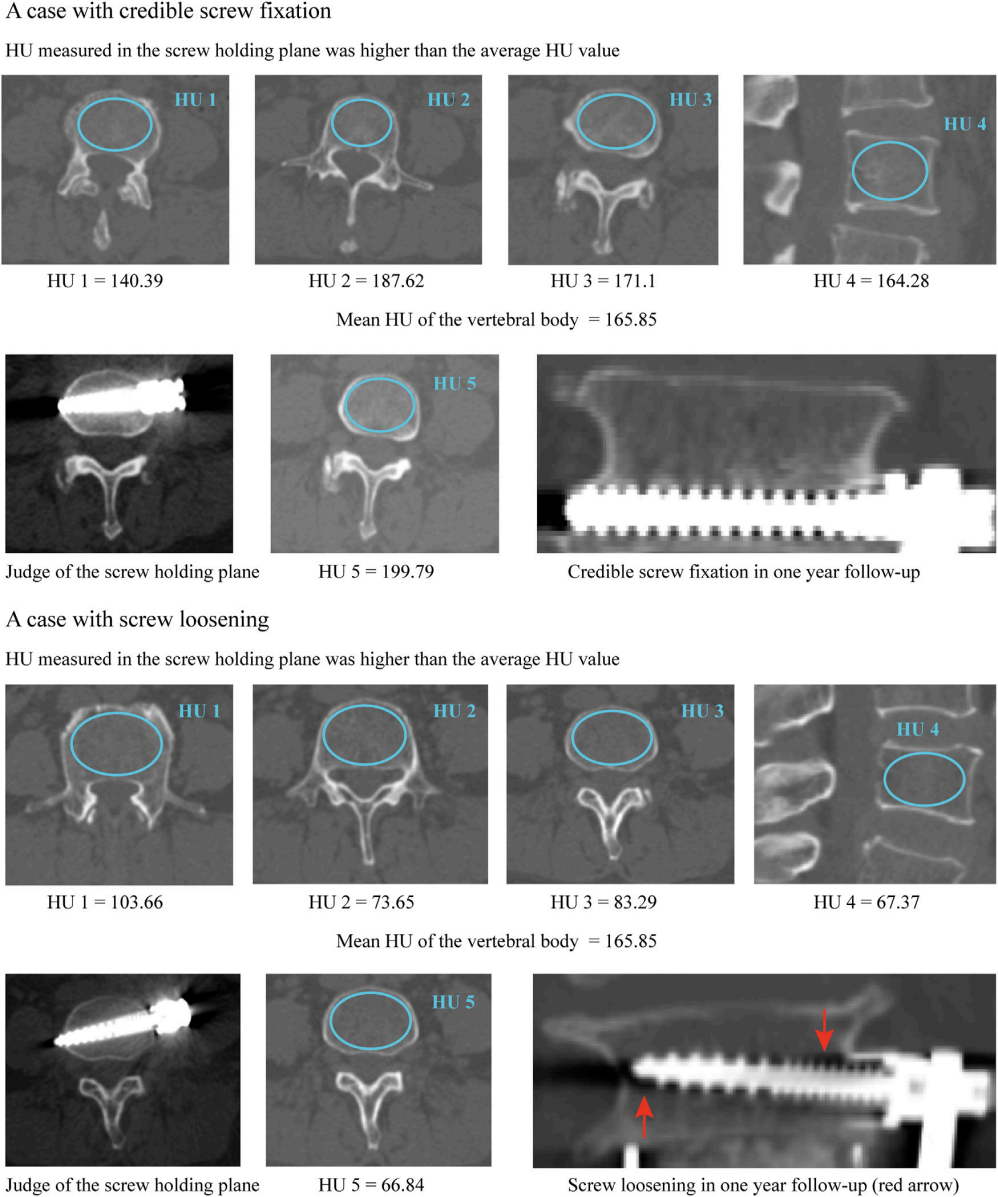
Typical cases for the better predictive performance of screw holding plane HU when predicting the risk of screw loosening.

The screw loosening status of the cranial and caudal vertebral bodies was judged separately. In the postoperative 1 year CT imaging data, vertebral bodies with ≥1 mm width radiolucent zones around the screw were defined as screw loosening (Figure 2) (Bredow et al., 2016; Bokov et al., 2019; Zou et al., 2020). The BMD of these patients was identified by measuring their Hounsfield unit (HU) values in the preoperative CT imaging data. During HU measurement in vertebral bodies, the region of interest was expanded to the largest within the cancellous bone but excluded other bony structures, such as **cortical shell**, BEP, and osteophytes (Schreiber et al., 2014; Xi et al., 2020; Zou et al., 2020). As mentioned above, HU was measured separately at the midsagittal plane, central transverse plane, and transverse planes close to the superior and inferior endplates. These HU values were defined as HU1 to HU4. The average value of these planes was set as the HU of the vertebral body (Pickhardt et al., 2013; Mikula et al., 2019; Xi et al., 2020; Zou et al., 2020). The screw holding plane was identified based on the instant postoperative CT imaging data (Ishikawa et al., 2018; Sakai et al., 2018; Xu et al., 2020). The HU value measured on the corresponding transverse plane in preoperative CT was defined as HU5 to represent the BMD of the screw holding cancellous bone (Figure 1).

Meanwhile, considering that disc distraction, segmental lordotic (SL) angle restoration, and cage position could affect local transmission patterns, these values were regarded as covariables and were also been measured in this study (Okuda et al., 2006; Landham et al., 2017). Disc height was measured on the central sagittal plane, and its value was defined as the average value of the anterior and posterior disc height. The difference in disc height between pre- and postoperation was defined as the value of disc distraction (Kaito et al., 2011; Havey et al., 2012). The SL angle was also measured on the central sagittal plane, and differences in SL between pre- and postoperation were defined as the value of SL restoration. The cage’s position was identified in the instant postoperative CT scan (Figure 1) (Labrom et al., 2005; Landham et al., 2017; Park et al., 2017; He et al., 2021).

#### 2.1.3 Statistical analyses

Radiographic and demographic indicators are presented as the mean ± standard deviation for continuous variables and the number (percentage) for categorical variables. We conducted statistical analyses in SPSS 23.0 software. The intraclass correlation efficiency (ICC) was computed to identify the repeatability of continuous variables (ICC ≥0.8 represents excellent reliability) (Zou et al., 2019; Zou et al., 2020). The kappa values were computed to determine the repeatability of screw loosening (kappa values of 0.41–0.60 indicated moderate reliability; 0.61–0.80, substantial agreement; and 0.81–1.00, excellent or almost perfect agreement) (Oetgen et al., 2008; Yue et al., 2008). ICC values were also computed to identify the consistency between HU values of the vertebral body and holding plane.

Statistical analyses for cranial and caudal side screw loosening were performed separately. When comparing the difference between different groups, the independent samples Student`s *t* test was used for continuous variables, and the chi-square test was used for the categorical variables. When comparing the significant difference between two groups by the Student’s *t*-test, all indexes from random samples were normal distribution, and all parameters of the experimental and control groups had homogeneity of variance. We performed binary logistic regression to identify independent risk factors for screw loosening. When using the binary logistic regression, the dependent variable (screw loosening status) is a binary classification variable (which is from different patients and therefore fully independent); its classification is complete and exclusive (screw loosening or not). In the multivariate analysis, all factors had no significant collinearity, and there are no obvious outliers and strong influence points for all included parameter values. Univariate analyses of each potential risk factor were performed, and the variables that achieved a significance level of *p* < 0.1 were entered into multivariate analyses. Variables with *p* < 0.05 were considered independent risk factors in the multivariate analyses (Zhao et al., 2009; Park et al., 2017; Bagheri et al., 2019; Pisano et al., 2020; Zou et al., 2020). Regarding the sample size in this study, we declare that this was a retrospective study, and all patients who meet the inclusive criteria were enrolled in it. In the multivariate analysis, the sample size is more than 20 times the number of independent variables. Therefore, we believe that the sample size in this study is sufficient to investigate potential risk factors for screw loosening. A *p* value less than 0.05 indicated a significant difference.

### 2.2 Numerical biomechanical simulations of changes in stress distribution

#### 2.2.1 Study design protocol of the surgical simulation

FEA is considered a reliable method for evaluating biomechanical changes related to screw loosening for its ability to accurately qualify the stress level of special components (Hsu et al., 2005; Kang et al., 2014; Guvenc et al., 2019). The most maligned limitation of numerical biomechanical simulations (i.e., FEA) is that FEA could not investigate the biomechanical significance of several covariables based on a single calibrated intact model. Adimittednly, the current FEA models have not simulated covariables, including SL restorations, cage positions, and disc distractions (i.e., changes in postoperative disc height) (Labrom et al., 2005; Kaito et al., 2010; Havey et al., 2012; Landham et al., 2017). Therefore, these models cannot identify the biomechanical significance of factors related to these covariables (e.g., changes in tensile stress of ligaments and muscles).

To demonstrate the reliability of numerical simulations (i.e., prove that the covariables mentioned above could not affect the screw loosening risk), these covariables have been included in regression analyses to judge the risk of screw loosening. Since these covariables did not differ significantly between the credible screw fixation group and the group of screw loosening and were not independent risk factors for screw loosening, we believe that not simulating these covariables will not affect the reliability of the numerical simulation results in this study. Therefore, when investigating the biomechanical significance of a particular variable, we believe researchers should eliminate the interference of covariables by reviewing radiographic and demographic data, and this may be a feasible method to optimize the credibility of FEA studies.

### 2.3 Model construction strategy

#### 2.3.1 Construction of the intact model

Simulation of OLIF with ALSR fixation was performed in a previously constructed and validated biomimetic lumbosacral FE model (L3-S1) (Li et al., 2021b; Xu et al., 2022b). Bone structures of the FE model include cortical shell, cancellous, and BEPs. The cortical thickness was set as 0.8 mm, and the thickness and morphology parameters (i.e., concave angles and depths) of BEPs were defined separately based on anatomic studies. Nonbony components include the intervertebral disc (IVD) and facet cartilages. The IVD consists of the nucleus, annulus, and cartilage endplates (CEPs). On the basis of imaging data measurements, the nucleus’s cross-sectional area accounted for 38% of the IVD (Li et al., 2021b). The annulus was divided into four different layers; the outline of the BEP covers the entire IVD, and that of the CEP covers the nucleus and inner part of the annulus (Jacobs et al., 2014; Delucca et al., 2016). Ligaments and facet capsules were defined as cable elements in the preprocessing process of finite element analysis (FEA) (Dreischarf et al., 2014; Li et al., 2021a). To optimize the computational accuracy of the FEA model, model calibration was performed by adjusting the annulus average radius and nucleus positions in our previously published studies (Li et al., 2021b; Xu et al., 2022b). Specifically, by repeatedly computing the range of motions (ROMs) in the L4-L5 segment and adjusting these calibrated parameters, the differences between computed ROMs and measured values from widely cited *in vitro* studies could be reduced, and the computational stress values can make a good representation of real biomechanical situations.

### 2.3.2 Construction of the OLIF model with ALSR fixation

The L4-L5 segment was selected to simulate ALSR fixed OLIF. Surgical simulations were performed based on a literature review and our surgical experience. In this process, lateral parts of the annulus, all of the nucleus, and CEPs were removed, and a polyether-ether-ketone (PEEK) OLIF cage (18 mm width and 50 mm length) filled with grafted bone was inserted into the interbody space (Guo et al., 2020; Xi et al., 2020). The lordotic angle and disc height of the postoperative models were identical to those of the intact model to eliminate the mechanical effects of these parameters (Kim et al., 2015b; Wang et al., 2019; Guo et al., 2020).

The three-dimensional model of the fixation screw was reversely constructed based on the outline of the screw used in ALSR fixation in our clinical practice. During the simulation of ALSR screw fixation, two titanium alloy screws were inserted into the L4-L5 vertebral bodies and penetrated the contralateral cortex. The axes of the screws in the transverse plane were parallel to the OLIF cage, whereas those in the coronal plane were parallel to the BEPs (Guo et al., 2020; Xie et al., 2020). Screw threads were preserved, and the screw compaction effect was simulated by adjusting the material property of cancellous around the thread (Hsu et al., 2005; Matsukawa et al., 2016). The connection between the screw tulip, the nut, and the spacer was simplified to increase the computational efficiency (Xu et al., 2022a).

### 2.4 Boundary and loading conditions

#### 2.4.1 Mesh generations and model validations

FEA in this study was performed in the “Ansys workbench 2020 r2 academic”. Hybrid elements (e.g., tetrahedron and hexahedron elements) with different sizes were set in different components of the FE model. Mesh refinement was set in structures with low thickness and large deformation (e.g., BEP, facet cartilage, posterior parts, and the outer layer of the annulus) (Chuang et al., 2013; Dreischarf et al., 2014; Xu et al., 2022b). To eliminate the confounding effect of mesh sizes on computational results, we performed a mesh convergence test on the calibrated intact model by evaluating the change in intradiscal pressure (IDP) with different mesh sizes. The model was considered converged if the change in the computed IDP was less than 3% (Ottardi et al., 2016; Fan et al., 2021). The degrees of freedom of S1 inferior surfaces were fixed entirely. Different directional moments were applied to the superior BEP of L3 (Delucca et al., 2016; Xu et al., 2022a). Additionally, we performed a multi-indicator model validation in the calibrated intact model. The computed ROM, IDP, disc compression, and facet contact force were compared within *in vitro* measured values (Wilson et al., 2006; Renner et al., 2007; Schilling et al., 2011). When the difference between the computed biomechanical value and the *in vitro* measured mean value is less than one standard deviation, the intact model is considered to be validated (Kim et al., 2013; Kim et al., 2015a; Kim et al., 2015b).

### 2.5 Material properties and contact types definition, and indicators selection.

In the definition of material properties (Table 1), **cortical shell** and cancellous bone were defined by anisotropic law. The annulus was assumed to be hypoelastic material, and the nucleus was set as a semifluid incompressible material (Li et al., 2021b; Xu et al., 2022b). The material properties of the surgical instrumented structure (i.e., PEEK and titanium alloy) were defined by isotropic law (Chuang et al., 2012; Hsieh et al., 2017). By defining the friction coefficients between different contact surfaces, stress levels immediately after operation were computed. Consistent with published studies, the contact between facet cartilages was set as frictionless. Moreover, given the screw loosening occurred in the short postoperative period, the instant postoperative biomechanical environment has been simulated by setting the frictional coefficient between BEP and GB as 0.46, and that between BEP and cage and screw-cancellous interfaces as 0.2 (Lu and Lu, 2019; Rastegar et al., 2020). The simulation of stepwise BMD reduction was performed by modifying the stiffness of bony tissues. In this process, the morphological features of different models remain identical. The material properties of bony tissues with different BMDs are presented in Table 1 (Morgan et al., 2003; Tsouknidas et al., 2015; Li et al., 2019). Finally, when it comes to the selection of computational indicators, the average stress of bone-screw interfaces and cancellous bone and the maximum stress of the screw could credibly judge changes in screw loosening risk (Tsuang et al., 2016; Pearson et al., 2017; Guvenc et al., 2019).

**TABLE 1:** Material properties of FE models’ components.

Components	Elastic modulus (MPa)	Poisson’s ratio	Cross-section (mm^2^)	References
Cortical (Normal BMD)	E_xx_ = 11,300	V_xy_ = 0.484		Ferguson and Steffen (2003), Tsouknidas et al. (2015)
	E_yy_ = 11,300	V_yz_ = 0.203		
	E_zz_ = 22,000	V_xz_ = 0.203		
	G_xy_ = 3,800			
	G_yz_ = 5,400			
	G_xz_ = 5,400			
Cancellous (Normal BMD)	E_xx_ = 140	V_xy_ = 0.45		Morgan et al. (2003), Tsouknidas et al. (2015)
	E_yy_ = 140	V_yz_ = 0.315		
	E_zz_ = 200	V_xz_ = 0.315		
	G_xy_ = 48.3			
	G_yz_ = 48.3			
	G_xz_ = 48.3			
Bony endplates (Normal BMD)	12,000	0.3		Li et al. (2019), Kang et al. (2014)
Cortical (Slight reduction of BMD)	Exx = 9,436	Vxy =0.484		Ferguson and Steffen (2003), Tsouknidas et al. (2015)
	Eyy = 9,436	Vyz =0.203		
	Ezz = 18,370	Vxz =0.203		
	Gxy = 3,173			
	Gyz = 4,509			
	Gxz = 4,509			
Cancellous (Slight reduction of BMD)	Exx = 93.8	Vxy = 0.45		Morgan et al. (2003), Tsouknidas et al. (2015)
	Eyy = 93.8	Vyz =0.315		
	Ezz = 150	Vxz =0.315		
	Gxy = 32.36			
	Gyz = 36.23			
	Gxz = 36.23			
Bony endplates (Slight reduction of BMD)	10,035	0.3		Li et al. (2019), Kang et al. (2014)
Cortical (Significant reduction of BMD)	Exx = 7,571	Vxy =0.484		Ferguson and Steffen (2003), Tsouknidas et al. (2015)
	Eyy = 7,571	Vyz =0.203		
	Ezz = 14,740	Vxz =0.203		
	Gxy = 2,546			
	Gyz = 3,618			
	Gxz = 3,618			
Cancellous (Significant reduction of BMD)	Exx = 47.6	Vxy = 0.45		Morgan et al. (2003), Tsouknidas et al. (2015)
	Eyy = 47.6	Vyz =0.315		
	Ezz = 100	Vxz =0.315		
	Gxy = 16.42			
	Gyz = 24.15			
	Gxz = 24.15			
Bony endplates (Significant reduction of BMD)	8,070	0.3		Li et al. (2019), Kang et al. (2014)
Annulus	Hypoelastic material			Kim et al. (2013), Wu and Yao (1976)
Nucleus	1	0.49		Chuang et al. (2013), Qasim et al. (2014)
Cartilage endplates	10	0.4		Li et al. (2019), Li et al. (2021a)
Anterior longitudinal ligaments	Calibrated load-deformation curved under different loading conditions	0.3	60	Du et al. (2016), Li et al. (2021b)
Posterior longitudinal ligaments	Calibrated load-deformation curved under different loading conditions	0.3	21	Du et al. (2016), Li et al. (2021a)
Ligamentum flavum	Calibrated load-deformation curved under different loading conditions	0.3	60	Du et al. (2016), Li et al. (2021b)
Interspinous ligaments	Calibrated load-deformation curved under different loading conditions	0.3	40	Du et al. (2016), Li et al. (2021a)
Supraspinous ligaments	Calibrated load-deformation curved under different loading conditions	0.3	30	Du et al. (2016), Li et al. (2021b)
Intertransverse ligaments	Calibrated load-deformation curved under different loading conditions	0.3	10	Du et al. (2016), Li et al. (2021a)
Capsular	7.5 (\25%)	0.3	67.5	Chuang et al. (2013), Li et al. (2019)
	32.9 ([25%)			
PEEK OLIF Cage	3500	0.3		Hsieh et al. (2017), Kang et al. (2014)
Titanium alloy screw	1,10,000	0.3		Hsieh et al. (2017), Kang et al. (2014)

## 3 Results

### 3.1 Retrospectively study of prospectively collected data

#### 3.1.1 Patient collection and screw loosening rates

**FIGURE 3:**
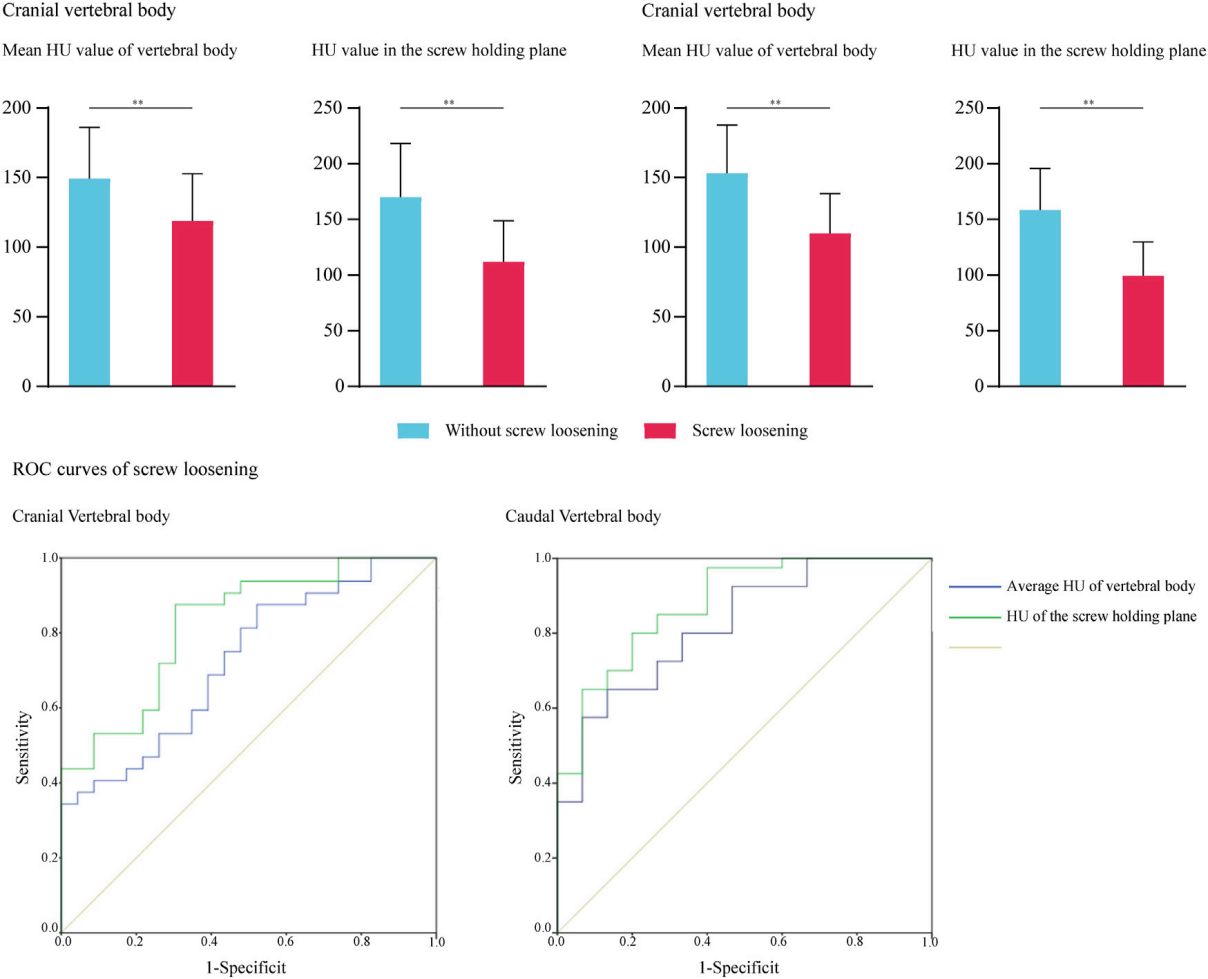
ROC curves for cranial and caudal side screw loosening and significant difference for HU in groups with credible screw fixation and screw loosening.

A total of 56 patients (30 males and 26 females) with an average age of 56.57 ± 11.96 years treated by single segment OLIF with ALSR screw fixation were recorded. The interobserver and intraobserver results during the judgment of screw loosening were substantial, with Kappa values of 0.778 and 0.759, respectively. The reliability of continuous variable measurement was excellent, with ICCs of 0.867 and 0.835, respectively (Table 2). The overall incidence rate of screw loosening was 35.71% (40/112), the screw loosening rate of the vertebral body on the cranial side was 42.86% (24/56) and that of the caudal vertebral body was 28.57% (16/56). There were no significant differences in HU between the cranial and caudal sides, whether the HU was measured by the mean value of vertebral bodies (*p* = 0.525) or in the screw holding plane (*p* = 0.707). Excellent consistency between vertebral bodies’ HU and HU of screw holding planes can be observed in cranial and caudal vertebral bodies and groups with credible screw fixation and screw loosening (Table 3). Although there were no significant differences, the HU of the screw holding planes was higher than the vertebral bodies’ HU in the credible screw fixation group and was lower than the mean HU of the vertebral bodies in the screw loosening group (Figures 2, 3).

**TABLE 2:** Validation of measured values repeatability.

	Interobserver	Intraobserver
ICCs of continuous variables	0.867	0.835
Kappa values of union status	0.789	0.746

**TABLE 3:** Validation of consistency between HU values of the vertebral body and holding plane.

	Credible screw fixation	Screw loosening
Cranial	0.897	0.958
Caudal	0.966	0.961

### 3.1.2 Identification of independent risk factors for screw loosening

The age of patients with cranial side screw loosening was significantly higher (*p* = 0.033). The HU values in the credible screw fixation group (i.e., without screw loosening) were significantly higher than those in the screw loosening group, whether the HU was measured by the mean value of vertebral bodies or in the screw holding plane (Figure 2). Based on the results of univariate logistic regression analyses, these three indicators were also entered into the multivariate analysis to identify independent risk factors. Considering the excellent consistency between vertebral bodies’ HU and HU of screw holding planes, the multivariate analysis of vertebral bodies’ HU and HU in screw holding planes was performed separately. The results showed that reducing HU, both measured by these two methods, was an independent risk factor for screw loosening on the cranial side (Tables 3, 4); the *p* value of vertebral bodies’ HU was 0.005, and that of HU in screw holding planes was 0.000.

**TABLE 4:** Logistic regression analysis of the cranial screw loosening.

	OR	95% CI		*p*
Univariate analysis				
Gender	2.333	0.791	6.885	0.125
Age	1.053	1.003	1.106	0.039^a^
BMI	0.972	0.83	1.138	0.723
SL restoration	1.1	0.949	1.275	0.208
Cage’s position	0.979	0.909	1.054	0.568
Disc distraction	1.152	0.829	1.601	0.399
HU (Mean value of vertebral body)	0.976	0.959	0.993	0.005^b^
HU (Screw holding plane)	0.969	0.952	0.986	0.000^b^
Multivariate analyses				
Age	1.038	0.984	1.095	0.172
HU (Mean value of vertebral body)	0.978	0.960	0.996	0.015^b^
Age	1.032	0.969	1.098	0.329
HU (Screw holding plane)	0.971	0.954	0.988	0.001^b^

a variables that achieved a significance level of *p* < 0.1 in the univariate analysis.

b statistical significance in the multivariate regression analysis (*p* < 0.05).

Concerning the caudal side, there were no significant age differences between patients with credible fixation and screw loosening (*p* = 0.117). The variation tendency of HU changes was consistent with the cranial vertebral body. Considering that only the *p* values of vertebral body HU and screw holding plane HU reduction were <0.1 in the univariate logistic regression analysis, multivariate analysis was not performed. The reduction of vertebral bodies’ HU and screw holding planes’ HU were regarded as independent risk factors for screw loosening in the caudal vertebral body (Figure 3 and Table 5); the *p* value of vertebral bodies’ HU was 0.001, of HU in screw holding planes, was 0.000, separately. Other covariables, including sex, BMI, SL restoration, disc distraction, and cage positions, did not significantly affect the risk of screw loosening. Additionally, the values of intraoperative covariables (i.e., cage position, SL restoration, disc distraction) were not significantly different between the credible screw fixation group and the screw loosening groups. These covariables were not independent risk factors for screw loosening on either the cranial or caudal sides.

**TABLE 5:** Logistic regression analysis of the caudal screw loosening.

	OR	95% CI		p
Univariate analysis				
Gender	1.739	0.54	5.604	0.354
Age	1.042	0.99	1.097	0.117
BMI	0.985	0.828	1.17	0.86
SL restoration	1.058	0.91	1.229	0.463
Cage’s position	0.986	0.91	1.068	0.734
Disc distraction	0.89	0.605	1.31	0.555
HU (Mean value of vertebral body)	0.957	0.933	0.982	0.001^b^
HU (Screw holding plane)	0.95	0.923	0.977	0.000^b^

b Statistical significance in the multivariate regression analysis (*p* < 0.05).

### 3.1.3 Parameter prediction values for screw loosening

We performed ROC curve analyses to assess the predictive value of vertebral body HU and HU measured in the screw holding plane; the results are summarized in Figure 3 and Table 6. Consistent with logistic regression analyses, HU values measured in the screw holding plane had the highest predictive ability. The area under the curves of screw holding plane HU in the cranial and caudal vertebral bodies were 0.828 and 0.88, respectively, and those of the vertebral body HU were 0.733 and 0.83, respectively. The sensitivity and specificity of the vertebral body’s HU were 0.875 and 0.5 in the cranial, 0.925 and 0.562 in the caudal vertebral body. The screw holding plane’s HU values were 0.875 and 0.652 in the cranial, 0.8 and 0.667 in the caudal vertebral body (Table 6).

**TABLE 6:** The cut-off value, sensitivity and specificity of four measurement methods for predicting screw loosening.

	Cut-off value	Sensitivity	Specificity	AUC
Cranial vertebral body				
HU (Mean value of vertebral body)	105.56	0.875	0.5	0.733
HU (Screw holding plane)	123.35	0.875	0.652	0.828
Caudal vertebral body				
HU (Mean value of vertebral body)	107.3	0.925	0.562	0.83
HU (Screw holding plane)	120.81	0.8	0.667	0.88

### 3.2 Numerical mechanical surgical simulations

#### 3.2.1 Multi-indicator model validation

Biomechanical indicators computed by the calibrated intact model were within ± 1 standard deviation of the average values measured by fresh specimens in widely cited *in vitro* studies. Thus, we believe that biomechanical changes identified by current FE models make good representations of actual stress levels (Figure 3).

### 3.2.2 Biomechanical changes caused by bone mineral density reductions.

**FIGURE 4:**
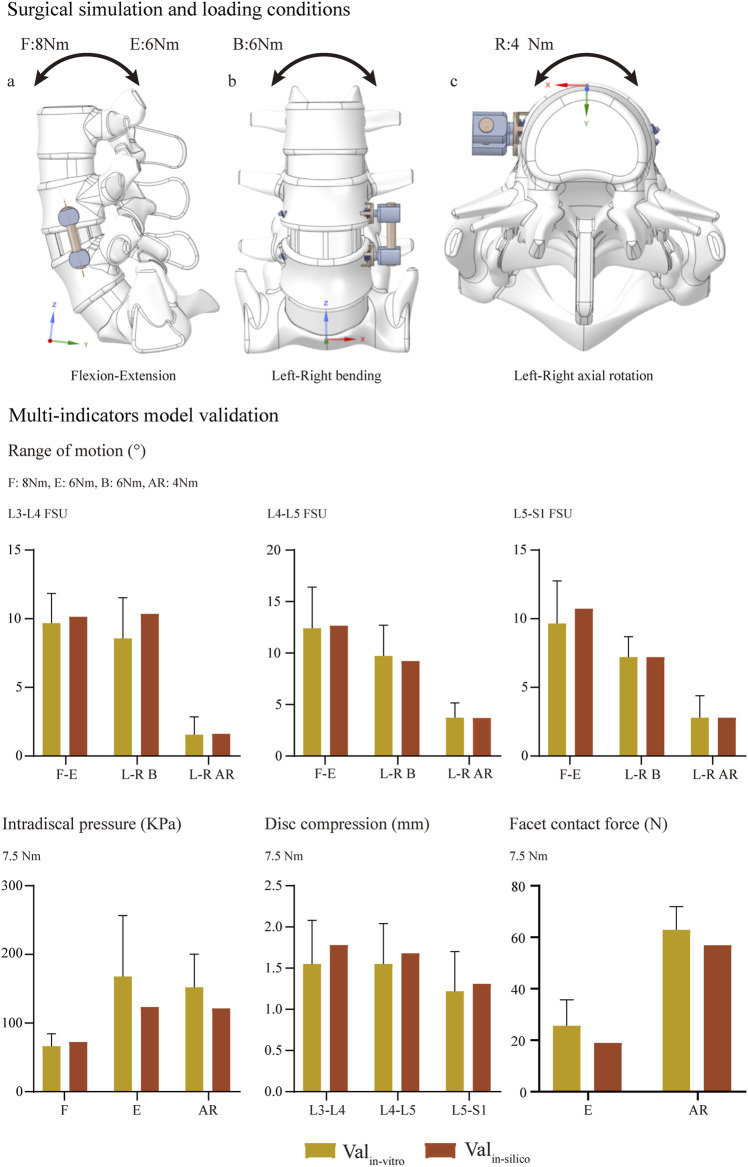
Surgical simulations and multi-indicator model validations (Wilson et al., 2006; Renner et al., 2007; Schilling et al., 2011).

Numerical simulations were performed under flexion, extension left and right bending, and axial rotation loading conditions (Figure 4). Loading conditions were identical to the calibration and validation of ROMs. Biomechanical changes in the cranial and caudal sides were computed separately. The maximum equivalent stress of screws and the average equivalent stress of bone-screw interfaces were computed and recorded to investigate local biomechanical changes in the screw holding position (Ambati et al., 2015; Matsukawa et al., 2016; Fletcher et al., 2019; Kim et al., 2020). The average equivalent stress of vertebral bodies was computed to investigate stress distribution changes (i.e., the proportion of load transportation in vertebral bodies and ALSR screw systems) in postoperative models with different BMDs.

**FIGURE 5:**
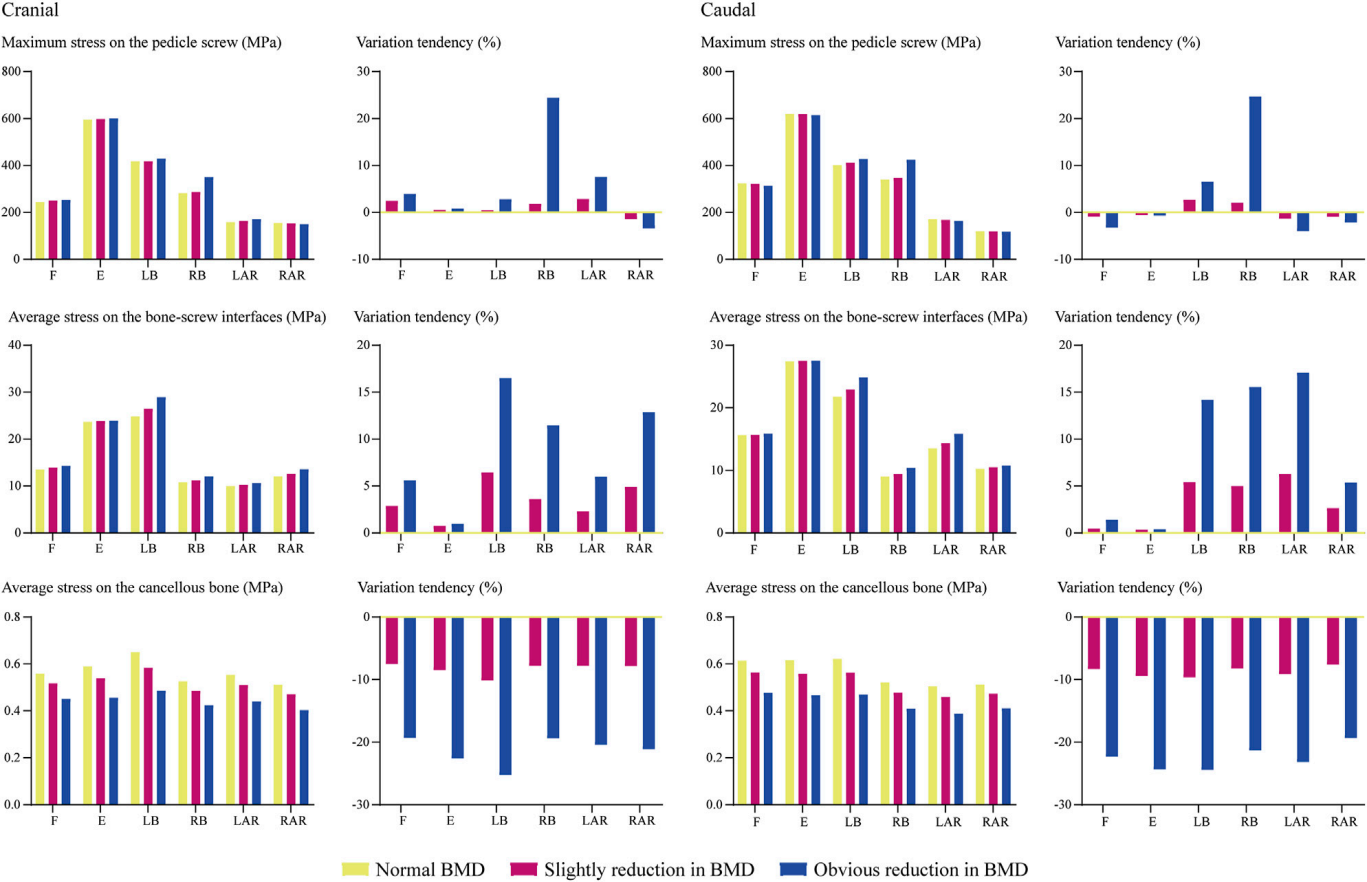
Changes in biomechanical indicators.

**FIGURE 6:**
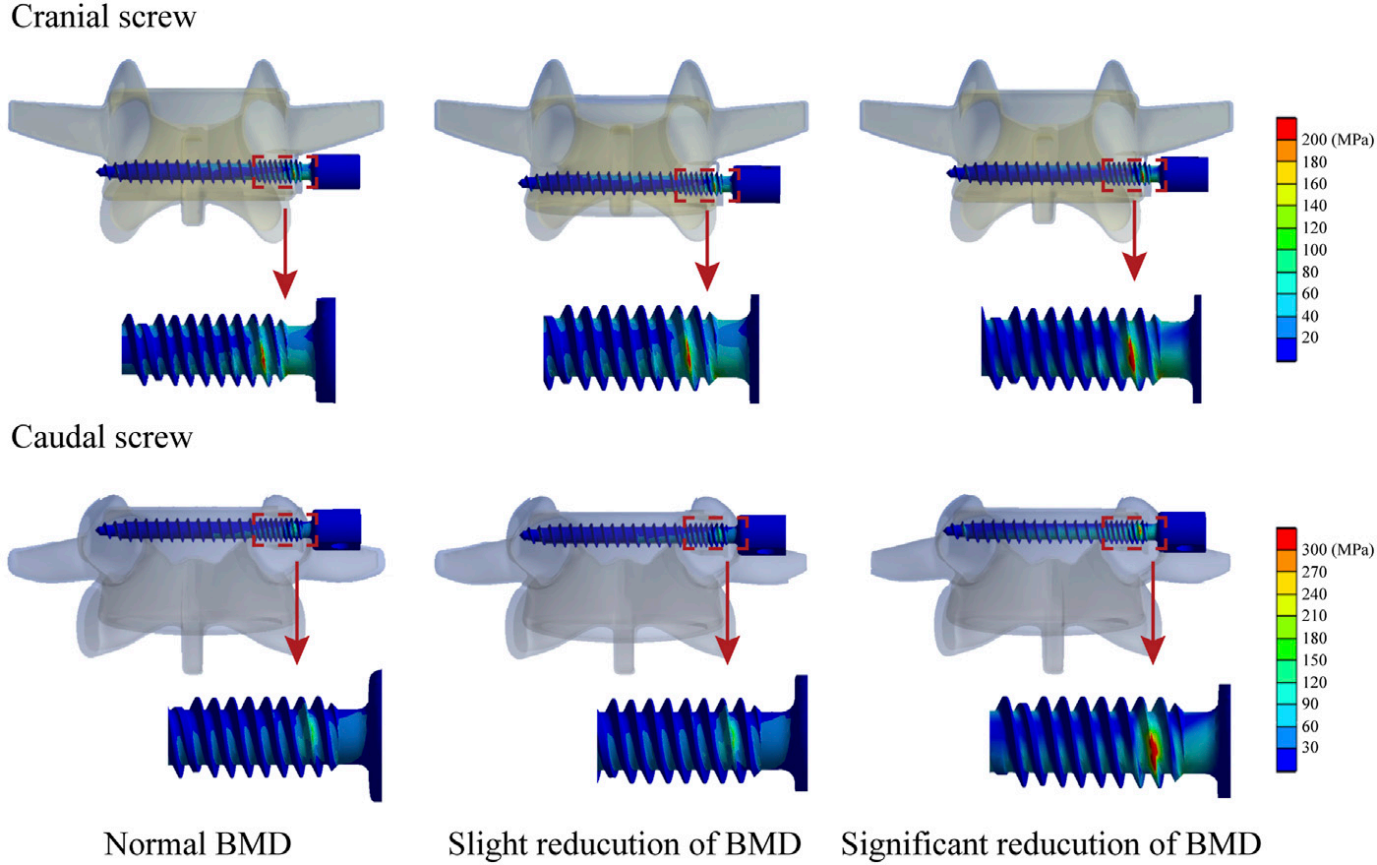
Nephograms for the maximum equivalent stress of screws under the right bending loading condition.

Changes in computed biomechanical indicators can explain the result from our radiographic and demographic data review. Consistent with published studies, stress concentration can be observed in the screw head of both cranial and caudal screws (Chao et al., 2008; Amaritsakul et al., 2014). With a stepwise reduction of BMD, higher equivalent stress of bone-screw interfaces can be observed under all loading conditions. The increase in the maximum equivalent stress of the fixation screw can be observed under bending and left lateral rotation loading conditions. A slight reduction (less than 5%) in the maximum stress of the cranial screw could only be observed under the right axial rotation loading condition with stepwise BMD reduction. In contrast, the increase in maximum stress can only be observed under bending loading conditions of the caudal screw. Additionally, with a stepwise reduction in BMD, the average equivalent stress of cancellous bones in the fixation segment was reduced step by step. In the model with slight BMD reduction, the average cancellous equivalent stress was reduced by nearly 10%, by higher than 20% in the model with significant BMD reduction (Figures 5, 6).

## 4 Discussion

Multiple studies have revealed that the incidence of screw loosening is high in patients with osteoporosis, and studies have proven that poor BMD of the fixation segment is an independent risk factor for screw loosening by measuring the HU values of vertebral bodies (Bredow et al., 2016; Pisano et al., 2020; Xu et al., 2020). Although few studies proved that the HU measured in the pedicle screw trajectories could make a credible prediction of screw loosening (Ishikawa et al., 2018; Sakai et al., 2018; Xu et al., 2020), no published studies identified the differences in predictive performance between HU measured by vertebral bodies and screw holding planes during the prediction of ALSR screw loosening. Meanwhile, mechanical tests identified that poor BMD would lead to loose bony yield strength and bone-screw integration (Bokov et al., 2019; Weidling et al., 2020). The resulting lower pullout strength can be recorded in pull-out tests with lower BMD (Hsu et al., 2005; Chao et al., 2008). However, whether the stress distribution changes with BMD reduction and whether this change will aggravate stress concentrations on fixation screws and bone-screw interfaces have not been verified.

This study investigates the predictive performance of HU measured in vertebral bodies and holding planes. A radiographic review of this study proved that HU values measured by these two methods were independent risk factors for screw loosening in both cranial and caudal vertebral bodies. Given that poor bony yield strength and the resulting loss of bone-screw integration are commonly accepted mechanisms of screw loosening in osteoporotic patients and can be well reflected by changes in HU values (Ishikawa et al., 2018; Nowak, 2019), high ICC values between vertebral bodies and screw holding planes HU identified excellent consistency between these values (Table 3). The excellent predictive performance of vertebral bodies HU on the risk of screw loosening was at least partly rooted in the excellent reflection of yield strength changes in screw holding positions.

Meanwhile, considering that regional differences in BMD and strength in cancellous bone exist (Smit et al., 1997; Wegrzyn et al., 2010), we believe HU measured in the screw holding plane can better reflect BMD reduction and related loss of bony yield strength and potential risk of bone screw integration. Consistent with this hypothesis, the predictive performance of screw holding plane HU was better than that of vertebral bodies (Figure 3 and Table 6). Given that the ALSR screw trajectory is highly adjustable, we believe that the trajectory optimization of ALSR screws based on preoperative HU measurement is feasible to optimize bone-screw integration and reduce screw loosening risk by optimizing the yield strength of screw holding positions under the premise of constant BMD in a particular osteoporotic patient.

Additionally, we verified that changes in the stress distribution in the fixation segment with BMD reduction would lead to a higher proportion of load transported by the ALSR system, resulting in higher screw and bone-screw interface stress, also initially triggering a higher risk of screw loosening. Therefore, the current results provide a new perspective for understanding the pathogenesis of screw loosening in patients with poor BMD. In other words, both reduction of screw holding position yield strength and deterioration of stress distributions were triggers for screw loosening in osteoporotic patients, and the optimization of these two factors should effectively reduce the risk of screw loosening. Regular anti-osteoporosis therapy could achieve both objectives by increasing BMD in the fixation segment (i.e., increasing the yield strength of screw fixation positions and optimizing stress distribution in the fixation segment by alleviating the pathological stress shielding effect) should be promoted in patients with lumbar screw fixation.

Changes in the fusion segment’s stress distribution with BMD stepwise reduction could also provide a reasonable explanation for the clinical phenomenon observed in the radiographic review. Specifically, the screw loosening rate was higher in the cranial vertebral body, but HU values measured in vertebral bodies and screw holding planes, as significant risk factors for screw loosening, were not significantly different. As computed by the current numerical simulations, although the variation tendency of stress levels in bone-screw interfaces and cancellous bones were identical in both cranial and caudal vertebral bodies, the maximum equivalent stress step increased with BMD reduction except for the right axial rotation loading condition. In contrast, the caudal screw stress only increased under bending loading conditions. In other words, the maximum stress increased in the cranial screw but decreased in the caudal screw under flexion, extension, and left axial rotation loading conditions. Given the exact relation between the maximum stress increase and the increase in screw loosening risk, we believe the current computational result explains the higher screw loosening risk of the ALSR fixation system in the cranial vertebral body.

To our knowledge, the most significant contribution of this study in methodology is the combination between the clinical review and numerical biomechanical simulations. Previously, these researches have been separately performed; clinical studies have observed a phenomenon without directly explaining its biomechanical mechanism; biomechanical studies have explored the potential mechanism of complications, but there is no clinical data to support this anticipation. Thus, the credibility of both types of studies is limited. In this study, the biomechanical mechanism of the observed clinical phenomenon has been directly investigated by corresponding numerical simulations. Combining these two parts is significant for better understanding a specific risk factor.

Admittedly, the current study results should be interpreted within the context of the following-mentioned limitations. Specifically, larger sample sizes of clinical data with a longer follow-up period should be obtained, and morphological changes during BMD reduction should be simulated in FEA models. However, given that screw loosening commonly occurs in the early stage of postoperative follow-up, the construction strategy of models with poor BMD has been widely reported (Ferguson and Steffen, 2003; Kealey et al., 2005; Zhang et al., 2010; Li et al., 2019), especially biomechanical changes that could well explain the result of clinical data observation. We believe the current study results are still reliable and could provide theoretical guidance for future clinical practice. Moreover, cortical bone is also important for screw fixation, but limited by the resolution of imaging data, we can not precisely measure the cortical thickness in current patients, and we admit that this is an important limitation for the study related to screw fixation strength evaluation. However, given the integration between cancellous bone and screw provide mainly screw holding strength, we believe the identification of cancellous BMD and corresponding biomechanical environment is still of great significance to deduce potential risk of screw loosening. Finally, for the lack of accurate references for the pretension of ALSR fixation, this factor has not been simulated in the current study, and we wish it can be accurately measured in our future studies.

## 5 Conclusion

Both vertebral bodies and “screw holding planes” HU can well predict screw loosening risk for OLIF patients with ALSR screw fixation. The predictive performance of screw holding plane HU is better than the mean HU of vertebral bodies. A higher proportion of ALSR load transmission triggers stress concentration on the screw and bone-screw interfaces in patients with poor BMD. This, together with decreased bony strength in the screw holding position, contributes to screw loosening in osteoporotic patients biomechanically. Therefore, the trajectory optimization of ALSR screws and regular anti-osteoporosis therapy may effectively reduce the risk of screw loosening.

## Data Availability

The raw data supporting the conclusions of this article will be made available by the authors, without undue reservation.

## Abbreviations

ALSR, anterior lateral single rod; BEP, bony endplate; BMD, bone mineral density; BMI, body mass index; CEP, cartilage endplate; CT, computational tomography; FEA, finite element analysis; HU, Hounsfield units; ICC, intraclass correlation efficiency; IDP, intradiscal pressure; IVD, intervertebral disc; OLIF, oblique lumbar interbody fusion; PEEK, polyether ether ketone; ROC, receiver operating characteristics; ROM, range of motion; SL, segmental lordotic.

## References

[B1] Agarwal A. Ingels M. Kodigudla M. Momeni N. Goel V. Agarwal A. K. (2016). Adjacent-level hypermobility and instrumented-level fatigue loosening with titanium and PEEK rods for a pedicle screw system: An *in vitro* study. J. Biomech. Eng. 138 (5), 051004. 10.1115/1.4032965 26974289

[B2] Amaritsakul Y. Chao C. K. Lin J. (2014). Biomechanical evaluation of bending strength of spinal pedicle screws, including cylindrical, conical, dual core and double dual core designs using numerical simulations and mechanical tests. Med. Eng. Phys. 36 (9), 1218–1223. 10.1016/j.medengphy.2014.06.014 25060212

[B3] Ambati D. V. Wright E. K. Jr. Lehman R. A. Jr. Kang D. G. Wagner S. C. Dmitriev A. E. (2015). Bilateral pedicle screw fixation provides superior biomechanical stability in transforaminal lumbar interbody fusion: A finite element study. Spine J. 15 (8), 1812–1822. 10.1016/j.spinee.2014.06.015 24983669

[B4] Bagheri S. R. Alimohammadi E. Zamani Froushani A. Abdi A. (2019). Adjacent segment disease after posterior lumbar instrumentation surgery for degenerative disease: Incidence and risk factors. J. Orthop. Surg. Hong. Kong) 27 (2), 2309499019842378. 10.1177/2309499019842378 31046589

[B5] Bokov A. Bulkin A. Aleynik A. Kutlaeva M. Mlyavykh S. (2019). Pedicle screws loosening in patients with degenerative diseases of the lumbar spine: Potential risk factors and relative contribution. Glob. Spine J. 9 (1), 55–61. 10.1177/2192568218772302 PMC6362557 30775209

[B6] Bredow J. Boese C. K. Werner C. M. Siewe J. Löhrer L. Zarghooni K. (2016). Predictive validity of preoperative CT scans and the risk of pedicle screw loosening in spinal surgery. Arch. Orthop. Trauma Surg. 136 (8), 1063–1067. 10.1007/s00402-016-2487-8 27312862

[B7] Chao C. K. Hsu C. C. Wang J. L. Lin J. (2008). Increasing bending strength and pullout strength in conical pedicle screws: Biomechanical tests and finite element analyses. J. Spinal Disord. Tech. 21 (2), 130–138. 10.1097/bsd.0b013e318073cc4b 18391719

[B8] Chuang W. H. Kuo Y. J. Lin S. C. Wang C. W. Chen S. H. Chen Y. J. (2013). Comparison among load-, ROM-, and displacement-controlled methods used in the lumbosacral nonlinear finite-element analysis. Spine 38 (5), E276–E285. 10.1097/brs.0b013e31828251f9 23250233

[B9] Chuang W. H. Lin S. C. Chen S. H. Wang C. W. Tsai W. C. Chen Y. J. (2012). Biomechanical effects of disc degeneration and hybrid fixation on the transition and adjacent lumbar segments. Spine 37 (24), E1488–E1497. 10.1097/brs.0b013e31826cdd93 22872225

[B10] DeLucca J. F. Cortes D. H. Jacobs N. T. Vresilovic E. J. Duncan R. L. Elliott D. M. (2016). Human cartilage endplate permeability varies with degeneration and intervertebral disc site. J. Biomechanics 49 (4), 550–557. 10.1016/j.jbiomech.2016.01.007 PMC4779374 26874969

[B11] Dreischarf M. Zander T. Shirazi-Adl A. Puttlitz C. M. Adam C. J. Chen C. S. (2014). Comparison of eight published static finite element models of the intact lumbar spine: Predictive power of models improves when combined together. J. Biomechanics 47 (8), 1757–1766. 10.1016/j.jbiomech.2014.04.002 24767702

[B12] Fan W. Guo L. X. Zhang M. (2021). Biomechanical analysis of lumbar interbody fusion supplemented with various posterior stabilization systems. Eur. Spine J. 30 (8), 2342–2350. 10.1007/s00586-021-06856-7 33948750

[B13] Ferguson S. J. Steffen T. (2003). Biomechanics of the aging spine. Eur. Spine J. 12 Suppl 2, S97–s103. 10.1007/s00586-003-0621-0 13680317 PMC3591832

[B14] Fletcher J. W. A. Windolf M. Grünwald L. Richards R. G. Gueorguiev B. Varga P. (2019). The influence of screw length on predicted cut-out failures for proximal humeral fracture fixations predicted by finite element simulations. Arch. Orthop. Trauma Surg. 139 (8), 1069–1074. 10.1007/s00402-019-03175-x 30895465

[B15] Gausden E. B. Nwachukwu B. U. Schreiber J. J. Lorich D. G. Lane J. M. (2017). Opportunistic use of CT imaging for osteoporosis screening and bone density assessment. J. Bone Jt. Surg. 99 (18), 1580–1590. 10.2106/jbjs.16.00749 28926388

[B16] Guo H. Z. Tang Y. C. Guo D. Q. Luo P. J. Li Y. X. Mo G. Y. (2020). Stability evaluation of oblique lumbar interbody fusion constructs with various fixation options: A finite element analysis based on three-dimensional scanning models. World Neurosurg. 138, e530–e538. 10.1016/j.wneu.2020.02.180 32156592

[B17] Guvenc Y. Akyoldas G. Senturk S. Erbulut D. Yaman O. Ozer A. F. (2019). How to reduce stress on the pedicle screws in thoracic spine? Importance of screw trajectory: A finite element analysis. Turk Neurosurg. 29 (1), 20–25. 10.5137/1019-5149.JTN.21895-17.2 29368324

[B18] Havey R. M. Voronov L. I. Tsitsopoulos P. P. Carandang G. Ghanayem A. J. Lorenz M. A. (2012). Relaxation response of lumbar segments undergoing disc-space distraction. Spine 37 (9), 733–740. 10.1097/brs.0b013e3182323adc 21912319

[B19] He L. Xiang Q. Yang Y. Tsai T. Y. Yu Y. Cheng L. (2021). The anterior and traverse cage can provide optimal biomechanical performance for both traditional and percutaneous endoscopic transforaminal lumbar interbody fusion. Comput. Biol. Med. 131, 104291. 10.1016/j.compbiomed.2021.104291 33676337

[B20] Hsieh Y. Y. Chen C. H. Tsuang F. Y. Wu L. C. Lin S. C. Chiang C. J. (2017). Removal of fixation construct could mitigate adjacent segment stress after lumbosacral fusion: A finite element analysis. Clin. Biomech. 43, 115–120. 10.1016/j.clinbiomech.2017.02.011 28259005

[B21] Hsieh Y. Y. Tsuang F. Y. Kuo Y. J. Chen C. H. Chiang C. J. Lin C. L. (2020). Biomechanical analysis of single-level interbody fusion with different internal fixation rod materials: A finite element analysis. BMC Musculoskelet. Disord. 21 (1), 100. 10.1186/s12891-020-3111-1 32059656 PMC7023693

[B22] Hsu C. C. Chao C. K. Wang J. L. Hou S. M. Tsai Y. T. Lin J. (2005). Increase of pullout strength of spinal pedicle screws with conical core: Biomechanical tests and finite element analyses. J. Orthop. Res. 23 (4), 788–794. 10.1016/j.orthres.2004.11.002 16022991

[B23] Ishikawa K. Toyone T. Shirahata T. Kudo Y. Matsuoka A. Maruyama H. (2018). A novel method for the prediction of the pedicle screw stability. Clin. Spine Surg. 31 (9), E473–e480. 10.1097/bsd.0000000000000703 30102636

[B24] Jacobs N. T. Cortes D. H. Peloquin J. M. Vresilovic E. J. Elliott D. M. (2014). Validation and application of an intervertebral disc finite element model utilizing independently constructed tissue-level constitutive formulations that are nonlinear, anisotropic, and time-dependent. J. Biomechanics 47 (11), 2540–2546. 10.1016/j.jbiomech.2014.06.008 PMC4366133 24998992

[B25] Kaito T. Hosono N. Fuji T. Makino T. Yonenobu K. (2011). Disc space distraction is a potent risk factor for adjacent disc disease after PLIF. Arch. Orthop. Trauma Surg. 131 (11), 1499–1507. 10.1007/s00402-011-1343-0 21706306

[B26] Kaito T. Hosono N. Mukai Y. Makino T. Fuji T. Yonenobu K. (2010). Induction of early degeneration of the adjacent segment after posterior lumbar interbody fusion by excessive distraction of lumbar disc space. Spi 12 (6), 671–679. 10.3171/2009.12.spine08823 20515354

[B27] Kang D. G. Lehman R. A. Jr. Bevevino A. J. Gaume R. E. Purcell R. L. Dmitriev A. E. (2014). Pedicle screw "hubbing" in the immature thoracic spine. J. Pediatr. Orthop. 34 (7), 703–709. 10.1097/bpo.0000000000000166 24590340

[B28] Kanno H. Aizawa T. Hashimoto K. Itoi E. (2021). Novel augmentation technique of percutaneous pedicle screw fixation using hydroxyapatite granules in the osteoporotic lumbar spine: A cadaveric biomechanical analysis. Eur. Spine J. 30 (1), 71–78. 10.1007/s00586-020-06451-2 32424638

[B29] Kealey S. M. Aho T. Delong D. Barboriak D. P. Provenzale J. M. Eastwood J. D. (2005). Assessment of apparent diffusion coefficient in normal and degenerated intervertebral lumbar disks: Initial experience. Radiology 235 (2), 569–574. 10.1148/radiol.2352040437 15798157

[B30] Kim H. J. Chun H. J. Lee H. M. Kang K. T. Lee C. K. Chang B. S. (2013). The biomechanical influence of the facet joint orientation and the facet tropism in the lumbar spine. Spine J. 13 (10), 1301–1308. 10.1016/j.spinee.2013.06.025 24035730

[B31] Kim H. J. Kang K. T. Chun H. J. Lee C. K. Chang B. S. Yeom J. S. (2015a). The influence of intrinsic disc degeneration of the adjacent segments on its stress distribution after one-level lumbar fusion. Eur. Spine J. 24 (4), 827–837. 10.1007/s00586-014-3462-0 25022861

[B32] Kim H. J. Kang K. T. Son J. Lee C. K. Chang B. S. Yeom J. S. (2015b). The influence of facet joint orientation and tropism on the stress at the adjacent segment after lumbar fusion surgery: A biomechanical analysis. Spine J. 15 (8), 1841–1847. 10.1016/j.spinee.2015.03.038 25817739

[B33] Kim H. Lee W. Choi S. Kholinne E. Lee E. Alzahrani W. M. (2020). Role of additional inferomedial supporting screws in osteoporotic 3-Part Proximal humerus fracture: Finite element analysis. Geriatr. Orthop. Surg. Rehabil. 11, 2151459320956958. 10.1177/2151459320956958 33224551 PMC7649924

[B34] Labrom R. D. Tan J. S. Reilly C. W. Tredwell S. J. Fisher C. G. Oxland T. R. (2005). The effect of interbody cage positioning on lumbosacral vertebral endplate failure in compression. Spine 30 (19), E556–E561. 10.1097/01.brs.0000181053.38677.c2 16205328

[B35] Landham P. R. Don A. S. Robertson P. A. (2017). Do position and size matter? An analysis of cage and placement variables for optimum lordosis in PLIF reconstruction. Eur. Spine J. 26 (11), 2843–2850. 10.1007/s00586-017-5170-z 28620787

[B36] Li J. Xu C. Zhang X. Xi Z. Liu M. Fang Z. (2021a). TELD with limited foraminoplasty has potential biomechanical advantages over TELD with large annuloplasty: An *in-silico* study. BMC Musculoskelet. Disord. 22 (1), 616. 10.1186/s12891-021-04504-1 34246272 PMC8272903

[B37] Li J. Xu C. Zhang X. Xi Z. Sun S. Zhang K. (2021b). Disc measurement and nucleus calibration in a smoothened lumbar model increases the accuracy and efficiency of *in-silico* study. J. Orthop. Surg. Res. 16 (1), 498. 10.1186/s13018-021-02655-4 34389025 PMC8362282

[B38] Li J. Xu W. Zhang X. Xi Z. Xie L. (2019). Biomechanical role of osteoporosis affects the incidence of adjacent segment disease after percutaneous transforaminal endoscopic discectomy. J. Orthop. Surg. Res. 14 (1), 131. 10.1186/s13018-019-1166-1 31088476 PMC6515674

[B39] Lu T. Lu Y. (2019). Comparison of biomechanical performance among posterolateral fusion and transforaminal, extreme, and oblique lumbar interbody fusion: A finite element analysis. World Neurosurg. 129, e890–e899. 10.1016/j.wneu.2019.06.074 31226452

[B40] Matsukawa K. Yato Y. Imabayashi H. Hosogane N. Abe Y. Asazuma T. (2016). Biomechanical evaluation of fixation strength among different sizes of pedicle screws using the cortical bone trajectory: What is the ideal screw size for optimal fixation? Acta Neurochir. 158 (3), 465–471. 10.1007/s00701-016-2705-8 26769471

[B41] Mi J. Li K. Zhao X. Zhao C. Q. Li H. Zhao J. (2017). Vertebral body hounsfield units are associated with cage subsidence after transforaminal lumbar interbody fusion with unilateral pedicle screw fixation. Clin. Spine Surg. 30 (8), E1130–e1136. 10.1097/bsd.0000000000000490 27906743

[B42] Mikula A. L. Puffer R. C. Jeor J. D. S. Bernatz J. T. Fogelson J. L. Larson A. N. (2019). Teriparatide treatment increases Hounsfield units in the lumbar spine out of proportion to DEXA changes. J. Neurosurg. Spine, 1–6. 10.3171/2019.7.SPINE19654 31628287

[B43] Morgan E. F. Bayraktar H. H. Keaveny T. M. (2003). Trabecular bone modulus-density relationships depend on anatomic site. J. Biomechanics 36 (7), 897–904. 10.1016/s0021-9290(03)00071-x 12757797

[B44] Nowak B. (2019). Experimental study on the loosening of pedicle screws implanted to synthetic bone vertebra models and under non-pull-out mechanical loads. J. Mech. Behav. Biomed. Mater. 98, 200–204. 10.1016/j.jmbbm.2019.06.013 31260911

[B45] Oetgen M. E. Yue J. J. la Torre J. J. Bertagnoli R. (2008). Does vertebral endplate morphology influence outcomes in lumbar total disc arthroplasty? Part II: Clinical and radiographic results as evaluated utilizing the vertebral endplate Yue-bertagnoli (VEYBR) classification. Int. J. Spine Surg. 2 (2), 101–106. 10.1016/sasj-2007-0119-rr PMC4365828 25802609

[B46] Okuda S. Oda T. Miyauchi A. Haku T. Yamamoto T. Iwasaki M. (2006). Surgical outcomes of posterior lumbar interbody fusion in elderly patients. J. Bone & Jt. Surg. 88 (12), 2714–2720. 10.2106/jbjs.f.00186 17142422

[B47] Ottardi C. Galbusera F. Luca A. Prosdocimo L. Sasso M. Brayda-Bruno M. (2016). Finite element analysis of the lumbar destabilization following pedicle subtraction osteotomy. Med. Eng. Phys. 38 (5), 506–509. 10.1016/j.medengphy.2016.02.002 26968784

[B48] Park S. J. Lee C. S. Chung S. S. Kang S. S. Park H. J. Kim S. H. (2017). The ideal cage position for achieving both indirect neural decompression and segmental angle restoration in lateral lumbar interbody fusion (LLIF). Clin. Spine Surg. 30 (6), E784–e790. 10.1097/bsd.0000000000000406 27352372

[B49] Pearson H. B. Dobbs C. J. Grantham E. Niebur G. L. Chappuis J. L. Boerckel J. D. (2017). Intraoperative biomechanics of lumbar pedicle screw loosening following successful arthrodesis. J. Orthop. Res. 35 (12), 2673–2681. 10.1002/jor.23575 28387967

[B50] Pickhardt P. J. Pooler B. D. Lauder T. del Rio A. M. Bruce R. J. Binkley N. (2013). Opportunistic screening for osteoporosis using abdominal computed tomography scans obtained for other indications. Ann. Intern Med. 158 (8), 588–595. 10.7326/0003-4819-158-8-201304160-00003 23588747 PMC3736840

[B51] Pisano A. J. Fredericks D. R. Steelman T. Riccio C. Helgeson M. D. Wagner S. C. (2020). Lumbar disc height and vertebral hounsfield units: Association with interbody cage subsidence. Neurosurg. Focus 49 (2), E9. 10.3171/2020.4.focus20286 32738808

[B52] Rastegar S. Arnoux P. J. Wang X. Aubin C. (2020). Biomechanical analysis of segmental lumbar lordosis and risk of cage subsidence with different cage heights and alternative placements in transforaminal lumbar interbody fusion. Comput. Methods Biomechanics Biomed. Eng. 23 (9), 456–466. 10.1080/10255842.2020.1737027 32169009

[B53] Renner S. M. Natarajan R. N. Patwardhan A. G. Havey R. M. Voronov L. I. Guo B. Y. (2007). Novel model to analyze the effect of a large compressive follower pre-load on range of motions in a lumbar spine. J. Biomechanics 40 (6), 1326–1332. 10.1016/j.jbiomech.2006.05.019 16843473

[B54] Sakai Y. Takenaka S. Matsuo Y. Fujiwara H. Honda H. Makino T. (2018). Hounsfield unit of screw trajectory as a predictor of pedicle screw loosening after single level lumbar interbody fusion. J. Orthop. Sci. 23 (5), 734–738. 10.1016/j.jos.2018.04.006 29866525

[B55] Schilling C. Krüger S. Grupp T. M. Duda G. N. Blömer W. Rohlmann A. (2011). The effect of design parameters of dynamic pedicle screw systems on kinematics and load bearing: An *in vitro* study. Eur. Spine J. 20 (2), 297–307. 10.1007/s00586-010-1620-6 21110209 PMC3030714

[B56] Schreiber J. J. Hughes A. P. Taher F. Girardi F. P. (2014). An association can be found between hounsfield units and success of lumbar spine fusion. HSS Jrnl 10 (1), 25–29. 10.1007/s11420-013-9367-3 PMC3903949 24482618

[B57] Smit T. H. Odgaard A. Schneider E. (1997). Structure and function of vertebral trabecular bone. Spine 22 (24), 2823–2833. 10.1097/00007632-199712150-00005 9431618

[B58] Tsouknidas A. Sarigiannidis S. O. Anagnostidis K. Michailidis N. Ahuja S. (2015). Assessment of stress patterns on a spinal motion segment in healthy versus osteoporotic bony models with or without disc degeneration: A finite element analysis. Spine J. 15 (3), S17–s22. 10.1016/j.spinee.2014.12.148 25576902

[B59] Tsuang F. Y. Chen C. H. Wu L. C. Kuo Y. J. Lin S. C. Chiang C. J. (2016). Biomechanical arrangement of threaded and unthreaded portions providing holding power of transpedicular screw fixation. Clin. Biomech. 39, 71–76. 10.1016/j.clinbiomech.2016.09.010 27693563

[B60] Wang W. T. Guo C. H. Duan K. Ma M. J. Jiang Y. Liu T. J. (2019). Dual pitch titanium-coated pedicle screws improve initial and early fixation in a polyetheretherketone rod semi-rigid fixation system in sheep. Chin. Med. J. Engl. 132 (21), 2594–2600. 10.1097/cm9.0000000000000335 31306218 PMC6846250

[B61] Wegrzyn J. Roux J. P. Arlot M. E. Boutroy S. Vilayphiou N. Guyen O. (2010). Role of trabecular microarchitecture and its heterogeneity parameters in the mechanical behavior of *ex vivo* human L3vertebrae. J. Bone Min. Res. 25 (11), 2324–2331. 10.1002/jbmr.164 PMC3179283 20564249

[B62] Weidling M. Oefner C. Schoenfelder S. Heyde C. E. (2020). A novel parameter for the prediction of pedicle screw fixation in cancellous bone - a biomechanical study on synthetic foam. Med. Eng. Phys. 79, 44–51. 10.1016/j.medengphy.2020.03.001 32197920

[B63] Wilson D. C. Niosi C. A. Zhu Q. A. Oxland T. R. Wilson D. R. (2006). Accuracy and repeatability of a new method for measuring facet loads in the lumbar spine. J. Biomechanics 39 (2), 348–353. 10.1016/j.jbiomech.2004.12.011 16321637

[B64] Xi Z. Mummaneni P. V. Wang M. Ruan H. Burch S. Deviren V. (2020). The association between lower Hounsfield units on computed tomography and cage subsidence after lateral lumbar interbody fusion. Neurosurg. Focus 49 (2), E8. 10.3171/2020.5.focus20169 32738801

[B65] Xie T. Wang C. Yang Z. Xiu P. Yang X. Wang X. (2020). Minimally invasive oblique lateral lumbar interbody fusion combined with anterolateral screw fixation for lumbar degenerative disc disease. World Neurosurg. 135, e671–e678. 10.1016/j.wneu.2019.12.105 31884124

[B66] Xu C. Huang C. Cai P. Fang Z. Wei Z. Liu F. (2022a). Biomechanical effects of pedicle screw positioning on the surgical segment in models after oblique lumbar interbody fusion: An *in-silico* study. Ijgm Vol. 15, 1047–1056. 10.2147/ijgm.s352304 PMC8818966 35140507

[B67] Xu C. Xi Z. Fang Z. Zhang X. Wang N. Li J. (2022b). Annulus calibration increases the computational accuracy of the lumbar finite element model. Glob. Spine J. 21925682221081224, 21925682221081224. 10.1177/21925682221081224 PMC10538312 35293827

[B68] Xu F. Zou D. Li W. Sun Z. Jiang S. Zhou S. (2020). Hounsfield units of the vertebral body and pedicle as predictors of pedicle screw loosening after degenerative lumbar spine surgery. Neurosurg. Focus 49 (2), E10. 10.3171/2020.5.focus20249 32738800

[B69] Yue J. J. Oetgen M. E. Jaramillo-de la Torre J. J. Bertagnoli R. (2008). Does vertebral endplate morphology influence outcomes in lumbar disc arthroplasty? Part I: An initial assessment of a novel classification system of lumbar endplate morphology. SAS J. 2 (1), 16–22. 10.1016/s1935-9810(08)70013-6 25802597 PMC4365656

[B70] Zhang L. Yang G. Wu L. Yu B. (2010). The biomechanical effects of osteoporosis vertebral augmentation with cancellous bone granules or bone cement on treated and adjacent non-treated vertebral bodies: A finite element evaluation. Clin. Biomech. 25 (2), 166–172. 10.1016/j.clinbiomech.2009.10.006 19917516

[B71] Zhao F. D. Pollintine P. Hole B. D. Adams M. A. Dolan P. (2009). Vertebral fractures usually affect the cranial endplate because it is thinner and supported by less-dense trabecular bone. Bone 44 (2), 372–379. 10.1016/j.bone.2008.10.048 19049912

[B72] Zhao L. Xie T. Wang X. Yang Z. Pu X. Lu Y. (2022a). Clinical and radiological evaluation of cage subsidence following oblique lumbar interbody fusion combined with anterolateral fixation. BMC Musculoskelet. Disord. 23 (1), 214. 10.1186/s12891-022-05165-4 35248042 PMC8898418

[B73] Zhao L. Xie T. Wang X. Yang Z. Pu X. Zeng J. (2022b2019). Whether anterolateral single rod can maintain the surgical outcomes following oblique lumbar interbody fusion for double-segment disc disease. Orthop surg, Zou D, Li W, deng C, du G, Xu NThe use of CT hounsfield unit values to identify the undiagnosed spinal osteoporosis in patients with lumbar degenerative diseases. Eur. Spine J. 28 (8), 1758–1766.

[B74] Zou D. Sun Z. Zhou S. Zhong W. Li W. (2020). Hounsfield units value is a better predictor of pedicle screw loosening than the T-score of DXA in patients with lumbar degenerative diseases. Eur. Spine J. 29 (5), 1105–1111. 10.1007/s00586-020-06386-8 32211997

