# Supplementary material for: Deterioration of the fixation segment’s stress distribution and the strength reduction of screw holding position together cause screw loosening in ALSR fixed OLIF patients with poor BMD
Source: Front Bioeng Biotechnol. 2022 Aug 30;10:922848. doi: 10.3389/fbioe.2022.922848 (PMC9468878; doi:10.3389/fbioe.2022.922848)
Supplement: Supplementary file 1 [file Table4.DOC]

**Table 4.** Logistic regression analysis of the cranial screw loosening.

|  | OR | 95% CI | | P |
| --- | --- | --- | --- | --- |
| Univariate analysis |  |  |  |  |
| Gender | 2.333 | 0.791 | 6.885 | 0.125 |
| Age | 1.053 | 1.003 | 1.106 | 0.039^#^ |
| BMI | 0.972 | 0.83 | 1.138 | 0.723 |
| SL restoration | 1.1 | 0.949 | 1.275 | 0.208 |
| Cage’s position | 0.979 | 0.909 | 1.054 | 0.568 |
| Disc distraction | 1.152 | 0.829 | 1.601 | 0.399 |
| HU （Mean value of vertebral body） | 0.976 | 0.959 | 0.993 | 0.005* |
| HU （Screw holding plane） | 0.969 | 0.952 | 0.986 | 0.000* |
|  |  |  |  |  |
| Multivariate analyses |  |  |  |  |
| Age | 1.038 | 0.984 | 1.095 | 0.172 |
| HU （Mean value of vertebral body） | 0.978 | 0.960 | 0.996 | 0.015* |
|  |  |  |  |  |
| Age | 1.032 | 0.969 | 1.098 | 0.329 |
| HU （Screw holding plane） | 0.971 | 0.954 | 0.988 | 0.001* |

^#^,variables that achieved a significance level of p < 0.1 in the univariate analysis

*, statistical significance in the multivariate regression analysis (P＜0.05)
