# Supplementary material for: Deterioration of the fixation segment’s stress distribution and the strength reduction of screw holding position together cause screw loosening in ALSR fixed OLIF patients with poor BMD
Source: Front Bioeng Biotechnol. 2022 Aug 30;10:922848. doi: 10.3389/fbioe.2022.922848 (PMC9468878; doi:10.3389/fbioe.2022.922848)
Supplement: Supplementary file 2 [file Table6.DOC]

**Table 6.** The cut-off value, sensitivity and specificity of four measurement methods for predicting screw loosening.

|  | Cut-off value | Sensitivity | Specificity | AUC |
| --- | --- | --- | --- | --- |
| Cranial vertebral body |  |  |  |  |
| HU （Mean value of vertebral body） | 105.56 | 0.875 | 0.5 | 0.733 |
| HU （Screw holding plane） | 123.35 | 0.875 | 0.652 | 0.828 |
|  |  |  |  |  |
| Caudal vertebral body |  |  |  |  |
| HU （Mean value of vertebral body） | 107.3 | 0.925 | 0.562 | 0.83 |
| HU （Screw holding plane） | 120.81 | 0.8 | 0.667 | 0.88 |
