# Supplementary material for: Deterioration of the fixation segment’s stress distribution and the strength reduction of screw holding position together cause screw loosening in ALSR fixed OLIF patients with poor BMD
Source: Front Bioeng Biotechnol. 2022 Aug 30;10:922848. doi: 10.3389/fbioe.2022.922848 (PMC9468878; doi:10.3389/fbioe.2022.922848)
Supplement: Supplementary file 3 [file Table3.DOC]

**Table 3.** Validation of consistency between HU values of the vertebral body and holding plane.

|  | Credible screw fixation | Screw loosening |
| --- | --- | --- |
| Cranial | 0.897 | 0.958 |
| Caudal | 0.966 | 0.961 |
