# Supplementary material for: Deterioration of the fixation segment’s stress distribution and the strength reduction of screw holding position together cause screw loosening in ALSR fixed OLIF patients with poor BMD
Source: Front Bioeng Biotechnol. 2022 Aug 30;10:922848. doi: 10.3389/fbioe.2022.922848 (PMC9468878; doi:10.3389/fbioe.2022.922848)
Supplement: Supplementary file 4 [file Table1.DOC]

**Table 1.** Material properties of FE models’ components

| Components | Elastic modulus (MPa) | Poisson's ratio | Cross-section (mm^2^) | References |
| --- | --- | --- | --- | --- |
| Cortical  (Normal BMD) | E_xx_=11300  E_yy_=11300  E_zz_=22000  G_xy_=3800  G_yz_=5400  G_xz_=5400 | V_xy_=0.484  V_yz_=0.203  V_xz_=0.203 |  | (Tsouknidas et al., 2015; Ferguson and Steffen, 2003) |
| Cancellous  (Normal BMD) | E_xx_=140  E_yy_=140  E_zz_=200  G_xy_=48.3  G_yz_=48.3  G_xz_=48.3 | V_xy_=0.45  V_yz_=0.315  V_xz_=0.315 |  | (Morgan et al., 2003; Tsouknidas et al., 2015) |
| Bony endplates  (Normal BMD) | 12000 | 0.3 |  | (Li et al., 2019b; Kang et al., 2017) |
| Cortical  (Slight reduction of BMD) | Exx=9436  Eyy=9436  Ezz=18370  Gxy=3173  Gyz=4509  Gxz=4509 | Vxy=0.484  Vyz=0.203  Vxz=0.203 |  | (Tsouknidas et al., 2015; Ferguson and Steffen, 2003) |
| Cancellous  (Slight reduction of BMD) | Exx=93.8  Eyy=93.8  Ezz=150  Gxy=32.36  Gyz=36.23  Gxz=36.23 | Vxy=0.45  Vyz=0.315  Vxz=0.315 |  | (Morgan et al., 2003; Tsouknidas et al., 2015) |
| Bony endplates  (Slight reduction of BMD) | 10035 | 0.3 |  | (Li et al., 2019b; Kang et al., 2017) |
| Cortical  (Significant reduction of BMD) | Exx=7571  Eyy=7571  Ezz=14740  Gxy=2546  Gyz=3618  Gxz=3618 | Vxy=0.484  Vyz=0.203  Vxz=0.203 |  | (Tsouknidas et al., 2015; Ferguson and Steffen, 2003) |
| Cancellous  (Significant reduction of BMD) | Exx=47.6  Eyy=47.6  Ezz=100  Gxy=16.42  Gyz=24.15  Gxz=24.15 | Vxy=0.45  Vyz=0.315  Vxz=0.315 |  | (Morgan et al., 2003; Tsouknidas et al., 2015) |
| Bony endplates  (Significant reduction of BMD) | 8070 | 0.3 |  | (Li et al., 2019b; Kang et al., 2017) |
| Annulus | Hypoelastic material | |  | (Kim et al., 2010; Wu and Yao, 1976) |
| Nucleus | 1 | 0.49 |  | (Chuang et al., 2013; Qasim et al., 2014) |
| Cartilage endplates | 10 | 0.4 |  | (Li et al., 2019b; Li et al., 2021) |
| Anterior longitudinal  ligaments | Calibrated load-deformation curved under different loading conditions | 0.3 | 60 | (Du et al., 2016; Li et al., 2021) |
| Posterior longitudinal  ligaments | Calibrated load-deformation curved under different loading conditions | 0.3 | 21 | (Du et al., 2016; Li et al., 2021) |
| Ligamentum flavum | Calibrated load-deformation curved under different loading conditions | 0.3 | 60 | (Du et al., 2016; Li et al., 2021) |
| Interspinous  ligaments | Calibrated load-deformation curved under different loading conditions | 0.3 | 40 | (Du et al., 2016; Li et al., 2021) |
| Supraspinous  ligaments | Calibrated load-deformation curved under different loading conditions | 0.3 | 30 | (Du et al., 2016; Li et al., 2021) |
| Intertransverse  ligaments | Calibrated load-deformation curved under different loading conditions | 0.3 | 10 | (Du et al., 2016; Li et al., 2021) |
| Capsular | 7.5 (\25%)  32.9 ([25%) | 0.3 | 67.5 | (Chuang et al., 2013; Li et al., 2019b) |
| PEEK OLIF Cage | 3500 | 0.3 |  | (Hsieh et al., 2017; Kang et al., 2017) |
| Titanium alloy screw | 110000 | 0.3 |  | (Hsieh et al., 2017; Kang et al., 2017) |
