# Supplementary material for: Deterioration of the fixation segment’s stress distribution and the strength reduction of screw holding position together cause screw loosening in ALSR fixed OLIF patients with poor BMD
Source: Front Bioeng Biotechnol. 2022 Aug 30;10:922848. doi: 10.3389/fbioe.2022.922848 (PMC9468878; doi:10.3389/fbioe.2022.922848)
Supplement: Supplementary file 5 [file Table5.DOC]

**Table 5.** Logistic regression analysis of the caudal screw loosening.

|  | OR | 95% CI | | P |
| --- | --- | --- | --- | --- |
| Univariate analysis |  |  |  |  |
| Gender | 1.739 | 0.54 | 5.604 | 0.354 |
| Age | 1.042 | 0.99 | 1.097 | 0.117 |
| BMI | 0.985 | 0.828 | 1.17 | 0.86 |
| SL restoration | 1.058 | 0.91 | 1.229 | 0.463 |
| Cage’s position | 0.986 | 0.91 | 1.068 | 0.734 |
| Disc distraction | 0.89 | 0.605 | 1.31 | 0.555 |
| HU （Mean value of vertebral body） | 0.957 | 0.933 | 0.982 | 0.001* |
| HU （Screw holding plane） | 0.95 | 0.923 | 0.977 | 0.000* |

^#^,variables that achieved a significance level of p < 0.1 in the univariate analysis

*, statistical significance in the multivariate regression analysis (P＜0.05)
