# Supplementary material for: Deterioration of the fixation segment’s stress distribution and the strength reduction of screw holding position together cause screw loosening in ALSR fixed OLIF patients with poor BMD
Source: Front Bioeng Biotechnol. 2022 Aug 30;10:922848. doi: 10.3389/fbioe.2022.922848 (PMC9468878; doi:10.3389/fbioe.2022.922848)
Supplement: Supplementary file 6 [file Table2.DOC]

**Table 2.** Validation of measured values repeatability.

|  | Interobserver | Intraobserver |
| --- | --- | --- |
| ICCs of continuous variables | 0.867 | 0.835 |
| Kappa values of union status | 0.789 | 0.746 |
